# Correlation between inflammatory markers and enhanced recovery after surgery (ERAS) failure in laparoscopic colectomy

**DOI:** 10.1007/s00595-024-02958-z

**Published:** 2024-11-20

**Authors:** Ji Hyeong Song, Yoonsoo Shin, Kyung Ha Lee, Ji Yeon Kim, Jin Soo Kim

**Affiliations:** 1 Department of Surgery, Inje University Haeundae Paik Hospital, Busan, Korea; 2 College of Health and Medical Sciences, Daejeon University, Daejeon, Korea; 3 Department of Surgery, College of Medicine, Chungnam National University, Daejeon, Korea; 4 Department of Surgery, Chungnam National University Sejong Hospital, Sejong, Korea

**Keywords:** Colon neoplasms, Enhanced recovery after surgery, Inflammatory markers, Laparoscopy

## Abstract

**Purpose:**

To evaluate inflammatory markers to identify patients at risk of enhanced recovery after surgery (ERAS) failure following laparoscopic colectomy.

**Methods:**

We included patients who underwent laparoscopic colectomy between September 2020 and February 2023. ERAS failure was defined as intolerance of a soft diet on postoperative day (POD) 2, postoperative stay > 7 days, or readmission within 30 days postoperatively. Inflammatory markers were analyzed immediately postoperatively and on POD 1 and 3. All patients were subjected to the ERAS protocol and divided into success and failure groups.

**Results:**

Data from 402 patients (success, 330; failure, 72) were analyzed. The neutrophil-to-lymphocyte ratio (*p* < 0.001), platelet-to-lymphocyte ratio (*p* = 0.004), monocyte-to-lymphocyte ratio (*p* = 0.041), and C-reactive protein-to-albumin ratio (CAR; *p* < 0.001) were elevated in the failure group on POD 3. The immediate postoperative CAR was higher in the failure group (*p* = 0.045). ERAS failure occurred more frequently in patients with body mass index < 20 (*p* < 0.001), right colon tumors (*p* = 0.012), and longer operative time (*p* < 0.001).

**Conclusions:**

This study demonstrated that inflammatory markers are associated with ERAS failure. Among the inflammatory markers, CAR might be the most potent indicator of ERAS failure following laparoscopic colectomy.

## Introduction

Enhanced recovery after surgery (ERAS) is a comprehensive approach to perioperative care that encompasses preoperative, intraoperative, and postoperative interventions that are strategically synchronized to improve postoperative recovery. ERAS has revolutionized surgical care by challenging conventional practices and replacing them with evidence-based strategies. Initially designed for colectomies, ERAS guidelines for perioperative care in elective colorectal surgery were updated in 2018 [1]. Additionally, the protocol has been adapted for various surgical procedures with continuous refinement and adaptation to evolving practices in the field [2].

A meta-analysis established that the ERAS protocol demonstrated superior effectiveness and safety in laparoscopic colorectal cancer surgery compared to traditional care [3, 4]. It has also been successfully implemented in the management of colorectal cancer patients older than 70 years of age, demonstrating its general safety and feasibility [5]. Good compliance with the ERAS protocol has been shown to be correlated with reduced postoperative recovery times and improved clinical outcomes [6, 7].

However, despite the widespread adoption of the ERAS protocol, a subset of patients experience unexpected postoperative complications and prolonged recovery, ultimately resulting in ERAS failure. Although the concept of ERAS failure is not universally defined, it often encompasses adverse outcomes such as prolonged hospitalization, increased postoperative morbidity, and delayed return to functional status. In a systematic review of ERAS failure following laparoscopic colorectal surgery, the definition of ERAS failure was mostly associated with prolonged postoperative hospitalization [8]. Because no clear definition exists, there are no confirmed predictors of ERAS failure. The early recognition of patients at higher risk of ERAS failure allows for tailored perioperative management strategies, potentially reducing adverse events and improving clinical outcomes. Thus, identifying the predictive factors for ERAS failure is crucial in colorectal surgery.

Inflammatory markers are molecules that are produced in response to tissue injury or inflammation. Monitoring these markers may provide valuable insights into the physiological response to surgical trauma and provide relevant data for assessing the short- and long-term outcomes of patients with colorectal cancer [9]. Commonly used inflammatory markers include the neutrophil–lymphocyte ratio (NLR), platelet-lymphocyte ratio (PLR), monocyte-lymphocyte ratio (MLR), and C-reactive protein-albumin ratio (CAR), all of which are elevated in the presence of an inflammatory response.

Despite accumulating evidence supporting the utility of inflammatory markers in predicting surgical outcomes, their specific roles in identifying ERAS failure remain relatively unexplored. This study aimed to evaluate inflammatory markers to identify patients at risk of enhanced recovery after surgery (ERAS) failure following laparoscopic colectomy. By evaluating these markers, we aimed to provide valuable insights into the early recognition and management of patients at risk for prolonged recovery within the ERAS paradigm.

## Methods

### Patients

We retrospectively reviewed the medical records of patients who underwent laparoscopic or robotic colectomy at our institution between September 2020 and February 2023. The exclusion criteria were open surgery, emergency surgery, palliative surgery, and combined resection, which affect postoperative outcomes. Patient data, including demographic information, operative findings, histopathological reports, laboratory findings, and postoperative outcomes, were extracted from electronic medical records. As laparoscopic colectomy is not typically associated with significant blood loss, intraoperative blood loss is not routinely monitored at our institution unless there are specific circumstances, such as massive bleeding. TNM staging was performed according to the 8th edition of the American Joint Committee on Cancer staging system [10].

### ERAS protocols and the definition of ERAS failure

In 2018, the ERAS Society established evidence-based recommendations for 24 perioperative care items in elective colorectal surgery [1]. Surgeons use these guidelines and adapt them to each institution’s situation; our institution implemented 17 items (Table 1). Seventeen ERAS items were followed for all included patients. ERAS failure was defined as any of the following three criteria: (1) intolerance of soft diet on postoperative day (POD) 2, (2) postoperative length of stay (LOS) > 7 days, or (3) readmission within 1 month after surgery.

**Table 1:** Enhanced recovery after surgery protocols applied in our institute

Period	Protocol
Preadmission	Preadmission information, education and counselling
	Preoperative medical risk assessment and optimization
Preoperative	Intravenous antibiotic infusion within 60 min before skin incision
	Preoperative fluid and electrolyte therapy
	200 ml of oral carbohydrates 2 h before induction of anesthesia
Intraoperative	Standard anesthetic protocol
	Intraoperative fluid and electrolyte therapy
	Preventing intraoperative hypothermia using air warming device
	Minimally invasive surgery including laparoscopic and robotic approaches
Postoperative	No routine use of nasogastric tubes
	Postoperative multimodal analgesia
	Mechanical thromboprophylaxis and injection of low molecular weight heparin
	Near-zero fluid and electrolyte balance
	Urinary catheter removal on POD 1
	Postoperative glycemic control (< 150 mg/dL)
	Early oral feeding (liquid diet on POD 1, soft diet on POD 2)
	Early mobilization

*POD* Postoperative day

### Inflammatory markers

Complete blood counts (CBCs) and blood chemistry tests were routinely conducted three times: immediately postoperatively, on POD 1, and on POD 3. A CBC provides information on white blood cells, segmented neutrophils, lymphocytes, monocytes, and platelets. Blood chemistry tests revealed albumin and C-reactive protein (CRP) levels. The NLR, PLR, MLR, and CAR were calculated.

### Statistical analysis

IBM SPSS Statistics (version 26, IBM Corp., Armonk, N.Y., USA) was used for all statistical analyses. Categorical variables were analyzed using the chi-square or Fisher’s exact test. Continuous variables were analyzed using Student’s *t*-test. Receiver operating characteristic (ROC) curves were generated for the operative time and inflammatory markers. P values of < 0.05 were considered to indicate statistical significance.

### Ethical considerations

Institutional review board (IRB) approval was obtained to access patient data for research purposes (IRB No. 2023–10-005–003). The requirement for informed consent was waived because of the retrospective design and the use of de-identified patient data.

## Results

### Patient characteristics

Data from 402 patients were analyzed, including 330 and 72 patients in the success and failure groups, respectively. The characteristics and perioperative outcomes of patients according to ERAS success or failure are presented in Table 2. ERAS failure was more common in patients with a body mass index (BMI) < 20 (*p* < 0.001), tumors in the right colon (*p* = 0.012), and patients undergoing right hemicolectomy (*p* = 0.001). The failure group exhibited a longer operative time (*p* < 0.001), delayed time to tolerate a soft diet (*p* < 0.001), and an extended postoperative LOS (*p* < 0.001).

**Table 2:** Characteristics and perioperative outcomes of patients according to success or failure of the enhanced recovery after surgery protocol

Variables	ERAS		*p* value
	Success (*n* = 330)	Failure (*n* = 72)	
Sex			
Male	142 (43.0)	31 (43.1)	0.997
Female	188 (57.0)	41 (56.9)	
Age (years)			
< 75	230 (69.7)	50 (69.4)	0.966
≥ 75	100 (30.3)	22 (30.6)	
Body mass index (kg/m^2^)			
< 20	32 (9.7)	21 (29.2)	< 0.001
≥ 20	298 (90.3)	51 (70.8)	
ASA PS classification			
1–2	243 (73.6)	52 (72.2)	0.806
3–4	87 (26.4)	20 (27.8)	
Tumor location			
Right colon	109 (33.0)	35 (48.6)	0.012
Left colon	221 (67.0)	37 (51.4)	
Tumor size (cm)			
< 4	170 (51.5)	42 (58.3)	0.294
≥ 4	160 (48.5)	30 (41.7)	
Minimally invasive surgery			
Laparoscopy	325 (98.5)	70 (97.2)	0.614
Robot	5 (1.5)	2 (2.8)	
Type of operation			
Right hemicolectomy	107 (32.4)	33 (45.8)	0.001
Transverse colectomy	2 (0.6)	2 (2.8)	
Left hemicolectomy	22 (6.7)	7 (9.7)	
Anterior resection	164 (49.7)	18 (25.0)	
Low anterior resection	34 (10.3)	11 (15.3)	
Hartmann’s operation	1 (0.3)	1 (1.4)	
Histology			
Well or moderately differentiated	293 (88.8)	63 (87.5)	0.217
Poorly differentiated or mucinous	22 (6.7)	8 (11.1)	
Others	15 (4.5)	1 (1.4)	
TNM stage			
0-I	117 (35.5)	27 (37.5)	0.722
II-III	203 (61.5)	44 (61.1)	
Non-cancerous lesion	10 (3.0)	1 (1.4)	
Preoperative CEA*			
< 5	236 (76.6)	48 (73.8)	0.633
≥ 5	72 (23.4)	17 (26.2)	
Operation time (min)	148.31 ± 35.41	173.67 ± 48.31	< 0.001
Time to tolerate a soft diet	2.0 ± 0.0	5.07 ± 11.12	< 0.001
Postoperative length of stay	5.87 ± 2.13	12.63 ± 12.66	< 0.001

Categorical variables are presented as *n* (%); continuous variables are presented as mean ± standard deviation

*ERAS* Enhanced recovery after surgery, *ASA* American society of anesthesiologists, *PS* Physical status, *TNM* Tumor node metastasis, *CEA* Carcinoembryonic antigen

^*^Excluding 29 patients without preoperative CEA data

### Inflammatory markers

**Fig. 1:**
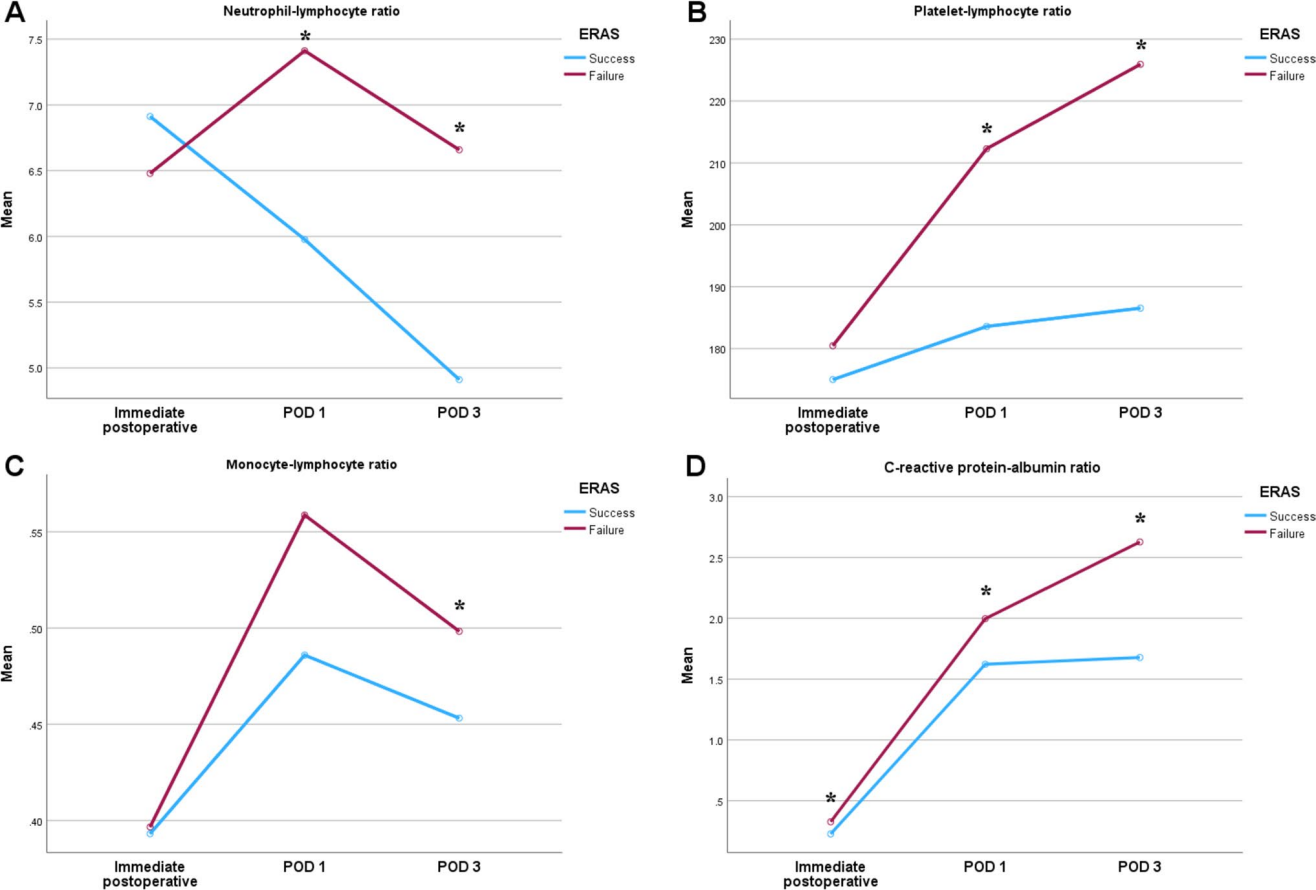
Differences in the mean values of inflammatory markers between the success and failure groups immediately after surgery, on postoperative day (POD) 1, and on POD 3. In the failure group, neutrophil–lymphocyte ratio (NLR) and platelet-lymphocyte radio (PLR) were significantly higher on POD 1 and 3. Monocyte-lymphocyte ratio (MLR) was significantly higher on POD 3. The C-reactive protein-albumin ratio (CAR) was significantly higher immediately after surgery than on POD 1 and 3. **A** NLR; **B** PLR; **C** MLR; **D** CAR. * *p* < 0.05

Table 3 shows the levels of inflammatory markers in the success and failure groups. On POD 3, the levels of all inflammatory markers, including NLR (*p* < 0.001), PLR (*p* = 0.004), MLR (*p* = 0.041), and CAR (*p* < 0.001), were higher in the failure group. Among them, NLR (*p* < 0.001), PLR (*p* = 0.013), and CAR (*p* = 0.004) were elevated on POD 1. In particular, the CAR immediately after surgery was significantly higher in the failure group (*p* = 0.045). Differences in inflammatory markers between the two groups are shown in Fig. 1.

**Table 3:** Laboratory findings according to success or failure of the enhanced recovery after surgery protocol

Variables	ERAS		*p* value
	Success (n = 330)	Failure (n = 72)	
White blood cell (10^3^/µL)			
Immediately after surgery	10.63 ± 3.45	11.11 ± 4.58	0.676
Postoperative day 1	8.58 ± 2.16	9.32 ± 3.06	0.068
Postoperative day 3	7.35 ± 1.85	8.05 ± 2.86	0.141
Platelet (10^3^/µL)			
Immediately after surgery	222.76 ± 63.17	238.22 ± 107.64	0.638
Postoperative day 1	208.98 ± 58.14	223.24 ± 97.14	0.711
Postoperative day 3	207.51 ± 59.96	218.17 ± 103.15	0.830
Segmented neutrophil (10^3^/µL)			
Immediately after surgery	8.45 ± 3.23	8.79 ± 3.89	0.686
Postoperative day 1	6.59 ± 2.05	7.51 ± 2.77	0.009
Postoperative day 3	5.29 ± 1.72	6.14 ± 2.42	0.014
Lymphocyte (10^3^/µL)			
Immediately after surgery	1.63 ± 0.88	1.70 ± 1.04	0.658
Postoperative day 1	1.30 ± 0.47	1.17 ± 0.48	0.025
Postoperative day 3	1.31 ± 0.47	1.15 ± 0.56	0.009
Monocyte (10^3^/µL)			
Immediately after surgery	0.51 ± 0.22	0.51 ± 0.23	0.600
Postoperative day 1	0.56 ± 0.19	0.55 ± 0.22	0.449
Postoperative day 3	0.49 ± 0.15	0.49 ± 0.26	0.172
Albumin (g/dL)			
Immediately after surgery	3.46 ± 0.39	3.41 ± 0.41	0.399
Postoperative day 1	3.27 ± 0.35	3.21 ± 0.34	0.221
Postoperative day 3	3.41 ± 0.36	3.28 ± 0.37	0.015
CRP (mg/dL)			
Immediately after surgery	0.73 ± 1.33	1.03 ± 1.41	0.030
Postoperative day 1	5.16 ± 2.67	6.33 ± 3.40	0.006
Postoperative day 3	5.52 ± 4.20	8.44 ± 5.40	< 0.001
Neutrophil–lymphocyte ratio			
Immediately after surgery	6.91 ± 4.84	6.48 ± 3.78	0.888
Postoperative day 1	5.99 ± 3.84	7.41 ± 4.84	< 0.001
Postoperative day 3	4.91 ± 6.25	6.66 ± 4.61	< 0.001
Platelet-lymphocyte ratio			
Immediately after surgery	175.06 ± 102.50	180.47 ± 118.57	0.917
Postoperative day 1	183.84 ± 91.21	212.29 ± 113.10	0.013
Postoperative day 3	186.54 ± 190.46	225.93 ± 147.48	0.004
Monocyte-lymphocyte ratio			
Immediately after surgery	0.39 ± 0.24	0.40 ± 0.32	0.424
Postoperative day 1	0.49 ± 0.25	0.56 ± 0.45	0.228
Postoperative day 3	0.45 ± 0.68	0.50 ± 0.32	0.041
CRP-albumin ratio			
Immediately after surgery	0.23 ± 0.45	0.33 ± 0.49	0.045
Postoperative day 1	1.62 ± 0.99	2.00 ± 1.10	0.004
Postoperative day 3	1.68 ± 1.40	2.63 ± 1.74	< 0.001

All variables are presented as mean ± standard deviation

*ERAS* Enhanced recovery after surgery, *CRP* C-reactive protein

### Predictors of ERAS failure

**Fig. 2:**
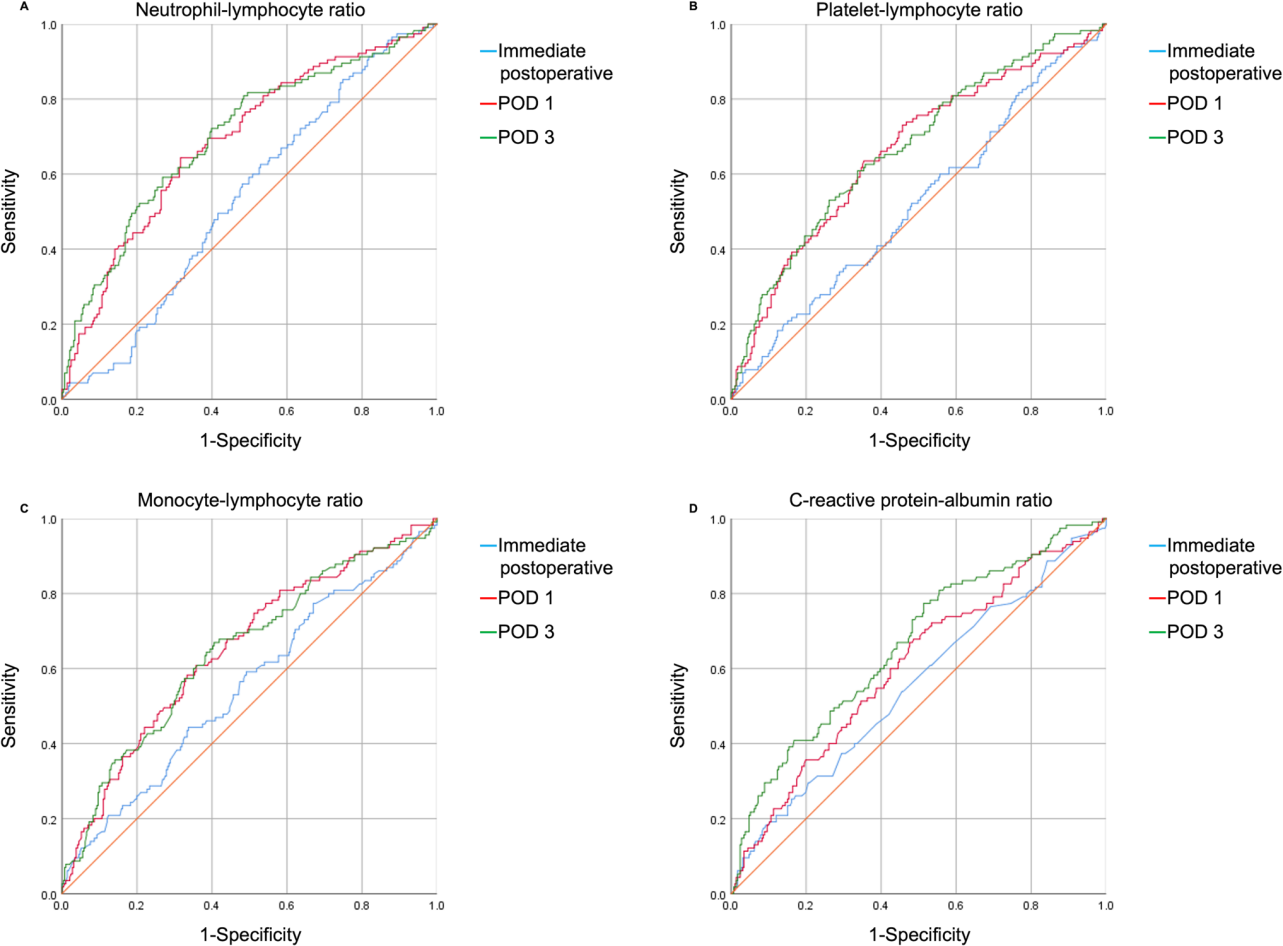
Receiver operating characteristic curves for inflammatory markers. The area under the curve values were highest on postoperative day 3 for all inflammatory markers. **A** Neutrophil–lymphocyte ratio; **B** platelet-lymphocyte radio; **C** monocyte-lymphocyte ratio; **D** C-reactive protein-albumin ratio. *POD* Postoperative day

**Fig. 3:**
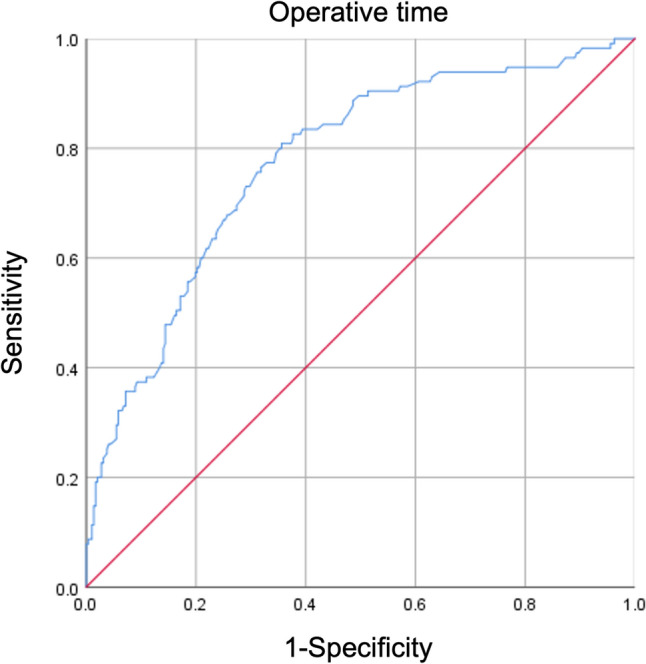
Receiver operating characteristic curves for operative time

The ROC curve analysis demonstrated the ability of inflammatory markers and operative time to predict ERAS failure (Figs. 2 and 3). The area under the curve (AUC) for operative time was 0.663, with a cutoff value of 160.5 min. Among the inflammatory markers, the CAR displayed the highest AUC, with values of 0.575 immediately after surgery and 0.684 on postoperative day (POD) 3. The cutoff values were 0.1737 immediately after surgery and 1.1664 on POD 3.

### Etiology of ERAS failure

All patients in the ERAS failure group experienced postoperative complications. Table 4 outlines the etiology of ERAS failure. The most common cause was a large amount of peritoneal drainage (defined as > 150 cc), followed by ileus. Severe complications (Clavien-Dindo classification ≥ III) accounted for approximately 7% of all complications. No mortality occurred within 1 month after surgery in any patient.

**Table 4:** Etiology of enhanced recovery after surgery failure (*n* = 72)

Etiology	*n* (%)
Large amounts of peritoneal drainage	31 (43.1)
Ileus	14 (19.4)
Bleeding	6 (8.3)
Anastomotic leakage	5 (6.9)
Atelectasis	4 (5.6)
Urinary tract infection	4 (5.6)
Chyle	2 (2.8)
COVID-19 infection	2 (2.8)
Diarrhea	2 (2.8)
Wound infection	1 (1.4)
Delirium	1 (1.4)

## Discussion

In the contemporary landscape of colorectal surgery, a paradigm shift toward minimally invasive surgery has become the standard, accompanied by widespread adoption of the ERAS protocol. The advantages of the ERAS protocol in colorectal surgery have been extensively documented [11–13] and, based on existing literature, include accelerated postoperative recovery, reduced morbidity, and improved clinical outcomes.

Despite the recognized benefits of the ERAS protocol, challenges persist in maintaining compliance, leading to instances of ERAS failure in clinical practice. Notably, the ERAS protocol may pose a risk to patients who are inadequately adapted to initiating a diet too early. This may result in an increased severity of complications and prolonged hospitalization. Recognizing the potential for ERAS failure and predicting at-risk patients is crucial. Identifying such cases upfront allows for judicious decision making, potentially opting for conventional care rather than ERAS protocols. Therefore, the present study sought to identify clinical characteristics and inflammatory markers as predictive indicators of ERAS failure.

Most previous studies defined ERAS failure as prolonged postoperative LOS [8]. However, it is not reasonable to define ERAS failure based on LOS because prolonged LOS may occur even in the absence of postoperative complications. Oh et al. [14] defined ERAS failure as readmission or mortality in addition to prolonged postoperative LOS. Stepwise dietary progression in the ERAS protocol after colorectal surgery is a critical component of a patient’s clinical course. In our ERAS protocol, we provided a liquid diet on postoperative day (POD) 1 and a soft diet on POD 2. Thus, intolerance to a soft diet on POD 2 was added to the definition of ERAS failure in this study.

Sun et al. [8] conducted a systematic review of ERAS failure and the associated risk factors after laparoscopic colorectal surgery. Their analysis, based on seven retrospective studies involving 1463 patients, identified 24 risk factors. Increased blood loss and longer operative times were the most prevalent, which aligns with these findings and indicates that prolonged operative time is a significant risk factor for ERAS failure. In addition to operative time, we investigated the potential predictive role of inflammatory markers in ERAS failure. Inflammatory markers have been widely studied in relation to postoperative complications in colorectal surgery [15, 16]. To the best of our knowledge, no previous study has analyzed the correlation between inflammatory markers and ERAS failure after laparoscopic colectomy. This study highlighted elevated NLR, PLR, MLR, and CAR as potential indicators of ERAS failure. Unlike other inflammatory markers, CAR demonstrated an immediate postoperative association, suggesting its potential role in decision-making in routine practice.

Higher AUC values indicate better diagnostic performance, with values exceeding 0.7 considered fair [17]. In our study, among the inflammatory markers, CAR on POD 3 showed the highest AUC value for predicting ERAS failure and operative time. However, since all AUC values in this study were < 0.7, they may not be reliable markers for predicting ERAS failure. Therefore, multiple clinical factors should be considered when predicting ERAS failure.

A study investigating the correlation between BMI and operative outcomes in major cancer surgeries revealed that patients classified as underweight (BMI < 18.5) exhibited worse outcomes [18]. Our study consistently demonstrated an association between low BMI and ERAS failure. This is likely attributable to the frequent coexistence of sarcopenia and malnutrition in underweight individuals. Future studies with preoperative assessments of sarcopenia and nutritional status would provide further support for this finding.

Garfinkle et al. [19] examined the association between right colectomy and the incidence of postoperative ileus in comparison to left colectomy. They found that the right colectomy group had higher rates of postoperative ileus, major morbidity, prolonged LOS, and 30-day readmission. Similarly, we observed a greater incidence of ERAS failure in right colectomy than in left colectomy. Further research is needed to understand the pathophysiological basis of why postoperative ileus is more frequent after right colectomy.

In this study, ileus was the second most common cause of ERAS failure. Implementing the ERAS protocol with early dietary initiation in patients at risk of postoperative ileus may prolong the LOS. Therefore, the early identification of these patients and selective application of the ERAS protocol would have a beneficial impact on postoperative recovery.

The present study was associated with several limitations. First, the retrospective nature of our investigation introduced a selection bias associated with data availability, and the single-center design may limit generalizability. Prospective multicenter studies with predefined protocols for the predictive role of inflammatory markers in ERAS failure are needed. Second, the definition of ERAS failure may not be completely inclusive. Our focus on postoperative LOS and dietary intolerance may have overlooked other factors that contributed to ERAS failure. Future studies should explore additional parameters such as patient quality of life and postoperative functional capacity to provide a more comprehensive assessment of ERAS failure. Finally, we routinely place drains after colectomy and tend to remove them late if the drainage volume is > 150 cc, which may lead to a prolonged LOS and act as a bias. The current ERAS guidelines recommend that peritoneal drains should not be routinely used, as they do not affect clinical outcomes.

In conclusion, this study demonstrated that inflammatory markers were associated with ERAS failure. Among the inflammatory markers, CAR might be the most potent indicator of ERAS failure following laparoscopic colectomy. By identifying specific risk factors and emphasizing the role of inflammatory markers, this study contributes to a nuanced understanding of ERAS strategies, facilitating enhanced patient care and improving surgical outcomes.
